# Unconscious Processing of Facial Emotional Valence Relation: Behavioral Evidence of Integration between Subliminally Perceived Stimuli

**DOI:** 10.1371/journal.pone.0162689

**Published:** 2016-09-13

**Authors:** Chengzhen Liu, Zhiyi Sun, Jerwen Jou, Qian Cui, Guang Zhao, Jiang Qiu, Shen Tu

**Affiliations:** 1 Department of Psychology, Institute of Education, China West Normal University, Nanchong 637002, China; 2 Faculty of Psychology, Southwest University, Chongqing 400715, China; 3 Department of Psychological Science, University of Texas—Rio Grande Valley, Edinburg, TX, 78539, United States of America; 4 School of Political Science and Public Administration, University of Electronic Science and Technology of China, Chengdu, 610054, China; 5 School of psychology, Liaoning Normal University, Dalian, 116029, China; Tilburg University, NETHERLANDS

## Abstract

Although a few studies have investigated the integration between some types of unconscious stimuli, no research has yet explored the integration between unconscious emotional stimuli. This study was designed to provide behavioral evidence for the integration between unconsciously perceived emotional faces (same or different valence relation) using a modified priming paradigm. In two experiments, participants were asked to decide whether two faces in the target, which followed two subliminally presented faces of same or different emotional expressions, were of the same or different emotional valence. The interstimulus interval (ISI) between the prime and the target was manipulated (0, 53, 163 ms). In Experiment 1, prime visibility was assessed post-experiment. In Experiment 2, it was assessed on each trial. Interestingly, in both experiments, unconsciously processed valence relation of the two faces in the prime generated a negative priming effect in the response to the supraliminally presented target, independent of the length of ISI. Further analyses suggested that the negative priming was probably caused by a motor response incongruent relation between the subliminally perceived prime and the supraliminally perceived target. The visual feature incongruent relation across the prime and target was not found to play a role in the negative priming. Because the negative priming was found at short ISI, an attention mechanism as well as a motor inhibition mechanism were proposed in the generation of the negative priming effect. Overall, this study indicated that the subliminal valence relation was processed, and that integration between different unconsciously perceived stimuli could occur.

## Introduction

Many studies have demonstrated that people can process unconscious information [1] and that the unconscious information can affect subsequent processing of conscious information [2,3]. In addition, the unconscious processing is not always automatic but can be modulated by top-down processes [4–6]. These results suggested that conscious and unconscious processes can influence each other.

The area in consciousness research that has not been systematically investigated but has great theoretical importance is the relationship between unconscious processes that can take place between different stimuli [7,8]. Thus far, preliminary studies [9–12] focusing on the relationship between unconscious processes occurring between different stimuli mainly investigated *integration* between different subliminally perceived stimuli. An unconscious integration was defined as a process that results in the generation of a new representation from unconscious processing of two or more component representations [13]. For example, using a go/no-go task and simultaneously presenting two shapes either supraliminally or subliminally, Lin and Murray [10] found that in the subliminal presentation condition the RT to the targets with same shape was significantly faster than to the targets with different shapes, suggesting that the *same-different relation* between the two subliminally perceived shapes could be processed. The *relation* between multisensory (e.g., audiovisual) stimuli [9] and between two digits or two letters [11,12] in the prime could also be processed unconsciously and influence the subsequent responses to the target. There is also evidence that gestalt-like configurations can be processed in the absence of consciousness [3,14].

As far as we know, there is little research investigating the integration between different subliminally perceived emotional stimuli. Many previous studies on unconscious emotional processing were concerned with the priming effect of a subliminal stimulus, and used a single emotional stimulus as a prime followed by a target. This is a serial presentation of a prime and a target [15,16]. A simultaneous presentation paradigm, in which the prime and the target are presented at the same time, was also used to evaluate the effect of an unconscious emotional stimulus on the target processing. For example, a subliminal facial expression was found to modulate the electrophysiological responses to a simultaneously presented supraliminal affective prosody [17]. Using go/no-go task, Tamietto and de Gelder [18] found that the responses to the conscious happy or fearful faces were modulated by the simultaneously presented subliminal emotional faces. Similar phenomena were observed in blindsight patients. For example, the emotional faces in the blind field influenced the behavioral responses to the same/different emotional faces simultaneously presented in the intact field [19,20]. Similarly, in some ERP and fMRI studies, it was found that the unseen emotional faces in the blind field could affect recognition of simultaneously presented voices [21,22] and visual encoding of seen emotional faces simultaneously presented in the intact field [23]. The unconscious emotion perception research and the relevant experimental paradigms were reviewed by Celeghin, Gelder & Tamietto [24] and Tamietto & de Gelder [25]. All these studies are concerned with the unconscious emotional *modulation* on conscious emotional processing. In addition, Carlson and Reinke [26] showed that a masked fearful face, which was simultaneously presented with a masked neutral face, could elicit spatial attention orientation and influence the following response to a dot-probe task. However, one aspect of the research these studies did not directly investigate is whether different unconsciously processed *emotional* stimuli could integrate with each other (i.e., generating a new representation). In an integration, two component representations fuse and generate a new representation.

In the present study, we used a priming paradigm to investigate whether an emotional valence relation between two unconsciously processed faces in a prime could influence the subsequent conscious processing of a pair of target faces. If we find a priming effect, the effect can be attributed to an integration of the two stimuli in the prime. It will also suggest that the unconsciously processed same-different emotional valence relation between the two faces in a prime can influence the subsequent conscious processing of emotional valence relation. Recently, using a masking paradigm and ERP and fMRI techniques, Tu, Martens, Zhao, Pan, Wang, Qiu, and Zhang [27] found that two simultaneously presented masked face pictures with different emotional valences, which was considered to be high-level complex information, elicited a smaller N2 and increased activation in the left middle frontal gyrus compared with two simultaneously presented masked face pictures with the same valence. They considered the results to reflect an unconscious mismatch detection (which, again, was high-level processing according to Mudrik, Faivre and Koch [13]) and suggested that an integration mechanism underlaid the unconscious processes. In another study, van Gaal et al. [28] found that multiple unconsciously perceived words could be integrated as indicated in an EEG recording. Consistent with these studies, Mudrik, Faivre & Koch [13] proposed that consciousness is not necessary for high-level integration over small temporal and spatial windows.

The first purpose of the present study was trying to extend the neural evidence of processing a relation between unconscious emotional valences in the Tu et al.’s [27] study to a behavioral level. In our experiments, two faces were subliminally presented side by side as a prime and followed by two target faces presented supraliminally side by side. The two faces in the pair of the prime and of the target showed either the same or different emotions. Participants were asked to judge whether the two target faces were of the same or different emotional valence. The valence relation (same or different) of the simultaneously presented faces was manipulated in both the prime and the target. If the same-different valence relation in the masked prime can affect the reaction time (RT) to the target, then that suggests that the emotional valence relation in the prime can be processed unconsciously and influence the subsequent conscious processing of facial emotional information.

Secondly, we examined possible prime/target visual congruency effect and motor response congruency effect through RT comparison between contrasting conditions. The priming effects of a masked prime on the target can result from a congruent versus an incongruent relation between the prime and the target in visual features (a *visual effect*) or a congruent versus an incongruent relation between an implicit response to the prime and an explicit response to the target (a *motor effect*) [29,30]. The visual effect can be measured by subtracting the RT to a target that is *visually congruent* with the prime from the RT to a target that is *visually incongruent* with the prime, while holding the response to the prime and the target constant. The motor response congruence effect can likewise be assessed by subtracting the RTs of an explicit response to a target that is *congruent with* the implicit response to the prime from the RT of an explicit response to a target that is *incongruent with* the implicit response to the prime, while holding the visual features constant. See detailed descriptions about the visual and motor effects of the experiments in “Visual effect” and “Motor response effect”.

Finally, three different interstimulus intervals (ISI) (0, 53, 163 ms) between the prime and the target were used to examine the role of the lengths of ISI in generating the positive and negative priming effects [29,30]. A negative priming is defined as a slowed response to a probe stimulus that is identical or related to an ignored prime stimulus relative to a control condition under a long ISI condition [31]. A positive priming effect typically appears when the prime and target are congruent and the ISI between them is short [32]. Based on these findings in the literature, we hypothesized that positive priming would occur under 0 ms and 53 ms ISI conditions, and negative priming under 163 ms condition. However, the results did not turn out the way as we predicted and will be reported later in the article.

It is crucial to insure that the perception of the masked stimuli in the prime was indeed unconscious. In this study, subjective measures such as PAS sale and objective measures such as forced-choice test were used to insure that the perception of the prime was unconscious [29,33]. Some studies assessed prime visibility after the main experiment [12,28] whereas others assessed it on each trial within the main experiment [29,33]. We conducted two experiments with visibility test conducted post-experiment in Experiment 1 and on each trial in Experiment 2.

## Experiment 1

The main purpose of Experiment 1 was to determine whether an unconsciously perceived relation between two masked facial emotional expressions in a prime could influence the RT for making a “same” or “different” decision for a pair of supraliminally perceived facial emotional expressions in a target stimulus that followed the masked prime. This effect was assessed by measuring the effects of visual and motor response congruence between the prime and the target. A post-experiment prime visibility test was conducted to assess the unconsciousness of the prime perception.

### Method

#### Participants

Ninety-six participants (69 women and 27 men) from China West Normal University volunteered for this experiment. A participant was randomly assigned to one of the three ISI conditions (0 ms, 53 ms and 163 ms). After excluding the participants who scored above chance level in the forced-choice visibility test, the remaining number of participants was 26 in the 0 ms ISI condition, 35 in the 53 ms ISI condition, and 31 in the 163 ms ISI condition. All participants were right-handed, had normal or corrected-to-normal vision, no history of, or current neurological or psychiatric illnesses. They gave their informed consent in writing before the experiment and were paid for their participation. This study was approved by the Institutional Review Board (IRB) of China West Normal University.

#### Materials

In this experiment, happy and fearful faces were used as stimuli. A sample consisting of 40 images of happy facial expressions (20 females, 20 males) and 40 images of fearful facial expressions (20 females, 20 males) was selected from the Chinese Facial Affective Picture System [34]. The scale for valence (from negative to positive emotion) ranged from 1 to 9 with 1 representing the most negative (least positive), and 9 most positive (least negative) emotion. Arousal scale (the intensity of the emotion) also ranged from 1 to 9 (extremely weak to extremely strong in arousal). The mean valence for the fearful faces was 2.73 (SD, 0.44) which was about the average for the negative half of the valence scale, and 6.28 (SD, 0.61) for the happy faces which corresponded to moderate happiness. The mean arousal for the fearful and happy faces was 6.31 (SD, 1.22) and 5.46 (SD, 1.17), respectively. During the experiment, two paired face images were displayed subliminally as the prime and supraliminally as the target side-by-side centrally on a uniform grey background and each face subtended approximately 4.3 (height) × 3.8 (width) degrees of visual angle. The four faces (two in the prime and two in the target) in each trial were faces of different people in all conditions.

#### Procedure

**Fig 1:**
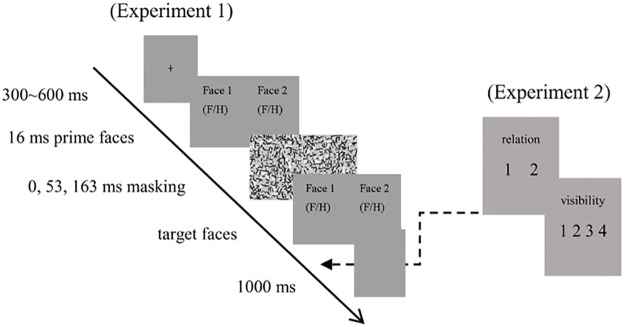
Schematic illustration of the sequentially displayed stimuli. Two masked paired-faces were displayed for 16 ms as a prime. Participants were asked to decide whether the two target faces were of the same valence or different valence. In experiment 1, prime visibility was assessed post-experiment. In Experiment 2, prime visibility was assessed on each trial. H represented happy facial expression and F represented fearful facial expression.

In the experiment, a fixation cross first appeared in the center of the screen for 300 to 600 ms with the exact duration randomly determined. Subsequently, a pair of faces serving as a prime was presented side-by-side on the screen for 16 ms, followed by a backward mask of two scrambled pictures for a duration of 53 or 163 ms. Then, another pair of faces, which was different from the two faces in the prime appeared as the target faces for these two ISI conditions. For the 0 ms ISI condition, there was no mask as such. The two target faces followed the prime immediately and served as both the mask and the target. The target faces were displayed and remained on the screen until participants responded. The participants were asked to decide whether the two test faces showed the same or different emotional expression by pressing “1” or “2” key with their right index and middle fingers, respectively. Finally, a blank screen appeared for 1000 ms. The sequence of events in a trail is displayed in Fig 1. The total 240 trials in each ISI condition were divided into two blocks to give participants a short between-blocks break. The different matching conditions in each block were presented in a random order.

After the participants finished the above phase of the experiment, they were asked to report whether or not they saw anything before the mask in order to assess the participants’ ability to recognize the masked pictures. After this self-report survey, participants were given a forced-choice discrimination task in which a masked face picture was followed by a pair of faces supraliminally presented on the left and right of the fixation cross, one of which had the same valence as the masked face and the other of a different valence. Participants were asked to determine which of these two faces showed the same valence as the earlier masked face. The two faces remained on the screen until the participant made a response. There were 80 trials for this discrimination task. Before performing this task, participants were informed that in each trial only the accuracy, and not the speed, of the response was important. The 163 ms ISI was not used in the forced-choice task, since the prime visibility level from this ISI was expected to be either the same as, or lower than that in the 53 ms ISI condition due to a longer masking for the 163 ms ISI condition.

Although the prime was strongly masked and the participants and the experimenters could not even sense any presence of object except the masking itself, the chance level performance in the forced-choice discrimination task could not rule out the possibility that the participants were aware of some parts of the masked face but were just unable to match the masked face valence with one of the two test faces. In order to insure that the masked faces were not consciously perceived in the prime, we recalled 32 available participants who participated in the original experiment to do another version of the forced-choice discrimination task.

The procedure was similar to that used in the formal experiment. Two paired faces appeared before a mask. In the 53 ms and 163 ms ISI conditions the mask was two scrambled pictures with a presentation duration of 53 ms and 163 ms, respectively and in the 0 ms ISI condition the mask was another two paired faces with a duration of 1100 ms (approximately the mean RT in the formal experiment). The participants were asked whether they saw the masked stimulus. If they said that they saw the masked stimulus, the next trial was started. If they said that they did not see the masked stimulus, two word choices (“face” and “tool”) rather than two pictures of faces appeared. Participants were informed that the probability of each of the two options (“face” and “tool”) being the masked stimulus was equal, and were asked to determine/guess which object was presented in the prime. The probability of the occurrence of each word on the left and right side was equal. The two options remained on the screen until the participant made a response. There were 60 trials for each ISI condition in this discrimination task. Before performing this task, participants were informed that in each trial only the accuracy, and not the speed, of the response was important. Each participant completed all three ISI conditions. After this task, the participants were asked to report their subjective experience, i.e., whether they could sense anything or any content of the masked stimulus.

#### Experimental design

Regarding the valence-relation matching between the prime and target, there were four conditions: SS, DD, SD and DS (Table 1). In this notation, the *left letter* stood for the valence relation of the two faces *in the prime* and the *right letter* stood for the valance relation of the two faces *in the target*. “S” stands for “same”, and “D” for “different”. For example, in “SS”, the first “S” denotes that the two faces in the prime display were matched in valence, i.e., either both happy or both fearful. DS denotes that the two faces in the prime had different valences and the two faces in the target have the same valence. Note that there were two subtypes in SS (SS_s_ and SS_c_) and in DD (DD_s_ and DD_c_), respectively (Table 1), which were used to assess the visual and motor effect. In the first subtype of SS, the “SS_s_” denote same emotional valence in the prime and the target (e.g., HH-HH meaning two happy faces in the prime and two happy faces in the target; or FF-FF meaning two fearful faces in the prime and two fearful faces in the target). Thus, in this subtype, both the visual features and the motor response were held constant across the prime and the target. In the second subtype of SS, the “SS_c_” denote opposite emotional valences (e.g., HH-FF or FF-HH) across the prime and the target. Thus, in this condition, the motor response was held constant (both were “same”), but the visual features of the two pairs of faces across the prime and the target were changed. Likewise, there were two subtypes of DD. In the first subtype DD_s_, both the visual features and the motor response were held constant (e.g., HF-HF or FH-FH). In the second subtype DD_c_, the motor response was held constant, but the visual features were changed across the prime and the target (e.g., HF-FH or FH-HF).

**Table 1:** The detail valence pairing information of each condition and the mean RTs±standard deviations (ms) as a function of ISI and prime-target congruence condition (overall congruency effect, visual and motor effects) in Experiment 1. Each of the column headings (e.g., SS, DD, DS, SD) denotes the valence relationship of the two faces, with the first letter denoting the valence relation of the two faces in the prime and the second letter that of the two faces in the target. S represented the same valences between two simultaneously presented face pictures and D represented different valences between two faces. H represented happy facial expression and F represented fearful facial expression. The valences of the two faces in the prime were denoted by the two left letters and those of the target were denoted by the two right letters. The visual features and the response were the same across prime and target in SS_s_ and DD_s_ conditions but the visual features changed in SS_c_ and DD_c_ conditions although the response did not change. The number in parentheses was the number of trials for each pairing condition.

Facial Emotional Valence Relation in Prime and Target. S = Same; D = Different.	SS		DD		DS	SD
	SS_s_	SS_c_	DD_s_	DD_c_		
Each Facial Emotion in Prime and Target. H = Happy; F = Fearful.	HH-HH(20)	HH-FF(20)	HF-HF(20)	HF-FH(20)	HF-HH(10)	HH-HF(10)
	FF-FF(20)	FF-HH(20)	FH-FH(20)	FH-HF(20)	HF-FF(10)	HH-FH(10)
					FH-HH(10)	FF-HF(10)
					FH-FF(10)	FF-FH(10)
0 ms ISI	1177.20±212.15		1294.67±225.03		1170.74±235.43	1254.02±205.60
	1184.20±238.32	1170.13±200.09	1291.98±228.80	1295.70±232.45		
53 ms ISI	1046.27±203.53		1134.34±183.72		1045.68±211.95	1122.78±188.40
	1033.08±192.26	1059.67±220.49	1130.53±173.23	1137.73±197.66		
163 ms ISI	1012.89±161.88		1089.02±145.07		997.78±162.97	1068.50±144.85
	1011.03±171.22	1014.68±156.13	1001.45±153.53	1079.92±141.80		

Under each of the prime-to-target valence relation transition types in Table 1, the *two pairs* of letters in each column denote the specific valence of the two faces in a pair in the prime (represented by the first pair of letters) and in the target (represented by the second pair of letters). For example, H denotes a happy facial expression and F a fearful facial expression. For each pair of faces in the prime or the target (e.g., HF), the *left letter* (H) indicated that a happy face was on the *left side of the fixation point* and the *right letter* (F) indicated that a fearful face was on the *right side of the fixation point*. The four sets of the 4-letter notation under each column heading denote all the possible face arrangements in the prime and target under that column heading.

In order to obtain 40 trials for each condition in the following visual and motor effect analysis, each pairing in Table 1 received different numbers of trials as shown in the parentheses following each pair of code letters. There were 240 trials for each ISI (0, 53, 163 ms) condition and the three ISI conditions were conducted between different participants.

### Results

#### Prime visibility test results

In the first forced-choice task, participants reported that they could not detect the masked faces and their valence. However, four participants (1 in 0 ms ISI, and 3 in 53 ms ISI) scored above chance level, with their mean percentage of correct recognition being above 58%, binomial test, p < .05. These participants’ data were excluded from further analyses. The remaining participants performed at chance level in the recognition task. For the 0 ms ISI condition, the mean percentage of correct recognition was 50.62%, not significantly different from chance level, *t* (25) = .538, *p* = .595, nor was the *d’* value (mean = .031, SE = .057) significantly different from zero, *t* (25) = .533, *p* = 0.599. For the 53 ms ISI condition, the mean percentage of correct recognition was 49.43%, not significantly different from chance level, *t* (34) = -.553, *p* = .584, nor was the *d’* value (mean = -.030, SE = .052) significantly different from zero, *t* (34) = -.568, *p* = .574.

In the second forced-choice task, each participant completed 180 trials in total. These trials were divided into 3 blocks of 60 trials each to give participants a short break between the blocks. In 51 of the total 96 blocks (32 participants with each completing 3 blocks), participants reported sensing no masked object throughout the 60 trials. In the remaining 45 blocks, the mean number of trials in which participants reported sensing the masked object was 2.0 (ranging from 1 to 5). However, post-experiment queries revealed that the reason in the few trials where participants reported sensing the masked object was not that the masked object was visible, but because they simply made random guesses. In all the 96 blocks, only three blocks (two in the 0 ms ISI, one in the 53 ms ISI) had a mean percentage of correct recognition above chance level. In the remaining 93 blocks, the correct recognition rate was not different from the chance level. For the 0 ms ISI condition, the mean percentage of correct recognition was 48.47%, not significantly different from 50%, *t* (31) = -1.436, *p* = .161, nor was the d’ value (mean = -.108, SE = .105) significantly different from zero, *t* (31) = -1.028, *p* = .312. For the 53 ms ISI condition, the mean percentage of correct recognition was 47.84%, not significantly different from 50%, *t* (31) = -1.925, *p* = .063, nor was the d’ value (mean = -.048, SE = .090) significantly different from zero, *t* (31) = -.535, *p* = .596. For the 163 ms ISI condition, the mean percentage of correct recognition was 48.50%, again, not significantly different from 50%, *t* (31) = -1.255, *p* = .219, nor was the d’ value (mean = -.113, SE = .092) significantly different from zero, *t* (31) = -1.219, *p* = .232. In addition, a one-way repeated measures ANOVA with ISI as a within-participants factor on recognition accuracy of the masked prime did not reveal a significant effect of ISI, *F* (2, 62) = .116, *p* = .891.

#### Overall congruency effect

To investigate the priming effect possibly caused by the unconsciously processed same-different valence relation in the prime, a 2 (valence relation between the two faces in prime: same versus different, a within-subjects factor) by 2 (valence relation between the two faces in target: same versus different, a within-subjects factor) by 3 (ISI: 0, 53 and 163 ms, a between-subjects factor) mixed factorial ANOVA was conducted. The dependent measure was RT for the correct same/different responses and the accuracy rates of the responses to the targets.

For the response accuracy, the ISI by prime valence relation by target valence relation 3-way interaction was not significant, *F* (2, 89) = .957, *p* = .388, η_p_^2^ = .021. The main effects of prime and target valence relationship were both nonsignificant, *F* (1, 89) = 1.927, *p* = .169, η_p_^2^ = .021 for the prime, and *F* (1, 89) = 1.157, p = .285, η_p_^2^ = .013 for the target, respectively, but the main effect of ISI was significant, *F* (2, 89) = 8.226, *p* = .001, η_p_^2^ = .156. The interaction between prime and ISI, and the interaction between target and ISI were both nonsignificant (all *Fs* < .796, all *ps* > .454). The interaction between the prime and the target was significant, *F* (1, 89) = 9.527, *p* = .003, η_p_^2^ = .097. The simple effect showed that: (1) when the target was of same valence, the response accuracy for the target was higher for the prime of different valence (mean = 93.40%) than for the prime of same valence (mean = 91.78%), *F* (1, 89) = 11.33, *p* = .001; (2) when the target was of different valence, the difference in accuracy between the prime of different valence (mean = 91.66%) and the prime of same valence (mean = 92.30%) was nonsignificant, *F* (1, 89) = 1.35, *p* = .248.

**Fig 2:**
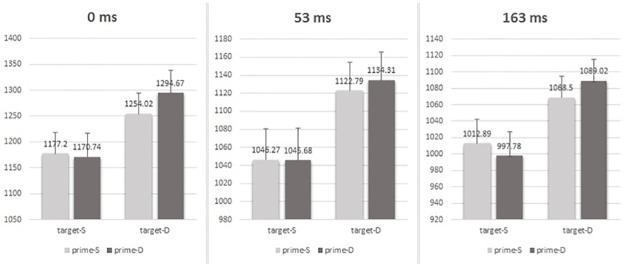
Mean RTs (ms) under each prime-target congruence condition (SS, SD, DS and DD) and each ISI (0ms, 53ms and 163ms). The three-way ANOVA analysis showed an interaction between prime and target. Further simple effects revealed that a negative priming occurred independent of three ISI conditions when the two faces in target were of different valence (for the target of same valence the negative priming was reflected in the response accuracy). Error bars represent the standard error of the mean.

For the RT results (see Table 1 and Fig 2), the ISI by prime valence relation by target valence relation 3-way interaction was not significant, *F* (2, 89) = 1.378, *p* = .257, η_p_^2^ = .030. The main effects of prime valence relation, target valence relation and ISI were all significant, *F* (1, 89) = 4.426, *p* = .038, η_p_^2^ = .047 for the prime, *F* (1, 89) = 102.257, *p* = .000, η_p_^2^ = .535 for the target, and *F* (2, 89) = 7.354, *p* = .001, η_p_^2^ = .142 for the ISI, respectively. The interaction between prime valence relation and target valence relation was also significant, *F* (1, 89) = 12.625, *p* = .001, η_p_^2^ = .124. But the interaction between prime valence relation and ISI, and the interaction between target valence relation and ISI were both nonsignificant (all *Fs* < 1.116, all *ps* > .332). The simple effects showed that (see Table 1): (1) when the target was of different valence, the RT for the prime of different valence (mean = 1164.37 ms) was slower than for the prime of same valence (mean = 1141.58 ms), *F* (1, 89) = 16.02, *p* = .000; (2) when the target was of same valence, the RT difference between the prime of different valence (mean = 1064.88 ms) and the prime of same valence (mean = 1072.026 ms) was nonsignificant, *F* (1, 89) = 1.56, *p* = .215.

In short, a negative priming was found in RT when the target was of different valence and in accuracy rates when the target was of same valence. In addition, the effect was independent of ISI conditions.

#### Visual effect

The effect of the visual-feature congruity relation between the prime and the target can be assessed by contrasting the RT to SSs with the RT to SSc (see Table 1). In this contrast, the motor responses were the same across the prime and the target. However, the valence of the pair of faces remained unchanged across the prime and the target in the SSs condition (e.g., HH-HH and FF-FF), but changed in the SSc condition (e.g., HH-FF and FF-HH). If RT difference between SSs and SSc conditions is observed, the difference reflects the effect of a visual or perceptual valence change from the prime to the target in the SSc condition relative to the SSs condition. Likewise, the visual effect could also be assessed by comparing RTs to DDs with RTs to DDc. A 2 (valence alteration across prime and target: unchanged versus changed) by 2 (valence relation between the two faces in the target: same versus different) by 3 (ISI: 0, 53 and 163 ms) mixed factorial ANOVA was conducted on RTs for the correct responses to assess the visual effect. There were 4 cells, i.e., SSs, SSc, DDs and DDc at each level of ISI. A significant visual effect would only reflect a prime/target visual congruence effect. It does not reflect the valence relation between the two simultaneously presented subliminal faces in the prime, therefore the visual effect is not our focus in this study.

**Fig 3:**
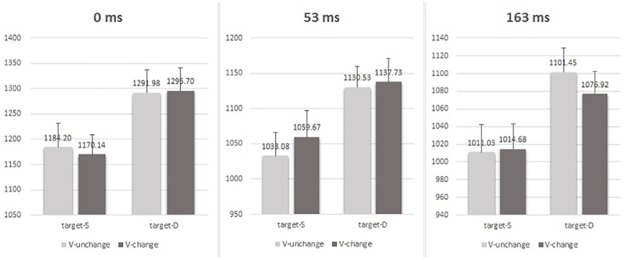
Mean RTs (ms) under each prime-target visual congruence condition (SS_s_, SS_c_, DD_s_ and DD_c_) and each ISI (0ms, 53ms and 163ms). The three-way ANOVA analysis did not reveal any visual effect. Error bars represent the standard error of the mean.

The ANOVA results (Table 1 and Fig 3) showed that none of the interaction effects was significant (all *Fs* < 2.195, all *ps* > .117). The main effect of target valence relation and ISI were both significant, *F* (1, 89) = 144.833, *p* = .000, η_p_^2^ = .619 for the target, and *F* (2, 89) = 7.627, *p* = .001, η_p_^2^ = .146 for the ISI, respectively. However, the main effect of visual change from prime to target was not significant, *F* (1, 89) = .005, *p* = .943, η_p_^2^ = .000. In short, these results suggested that the visual feature change probably did not contribute to the above negative priming effect.

#### Motor response effect

The motor response effect can be assessed by comparing RTs to DS with RTs to SSc and by comparing RTs to SD with RTs to DDc (see Table 1). In the contrast of DS versus SSc and of SD versus DDc, the visual features across the prime and the target were all changed but the motor responses changed only in the DS and SD. A 2 (response congruence across prime and target: congruent versus incongruent) by 2 (valence relation between the two faces in the target: same versus different) by 3 (ISI: 0, 53 and 163 ms) mixed factorial ANOVA was conducted on the RTs for the correct responses. At each level of ISI, there were four cells: SSc, DDc, SD and DS generated by the crossing of the two within-participants factors. A possible significant motor effect would reflect the integration between the two unconsciously perceived emotional faces.

**Fig 4:**
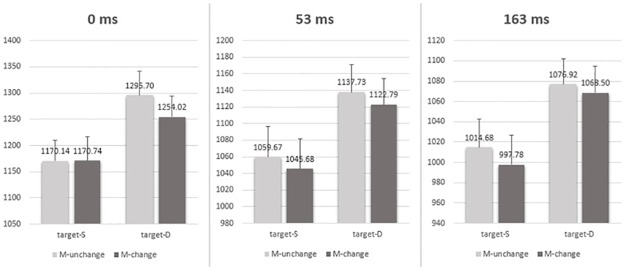
Mean RTs (ms) under each prime-target motor congruence condition (DS, SS_c_, SD and DD_c_) and each ISI (0ms, 53ms and 163ms). The three-way ANOVA analysis showed that the RTs to the changed response conditions (SD and DS) were faster than the RTs to the unchanged response conditions (DD_c_ and SS_c_) regardless of three ISI conditions. Error bars represent the standard error of the mean.

The ANOVA results (see Table 1 and Fig 4) showed that the main effect of target valence relation, motor changes and ISI were all significant, *F* (1, 89) = 88.779, *p* = .000, η_p_^2^ = .499 for the target valence relation, *F* (1, 89) = 10.301, *p* = .002, η_p_^2^ = .104 for the motor response change, and *F* (2, 89) = 7.213, *p* = .001, η_p_^2^ = .139 for the ISI, respectively. None of the interaction effects was significant (all *Fs* < 2.179, all *ps* > .119). These effects showed that the RTs to the motor response changed conditions (SD and DS) were faster than the RTs to the response unchanged conditions (DD_c_ and SS_c_). In short, the motor effect indicated a negative priming effect independent of ISI conditions.

## Experiment 2

One concern about the visibility test used in Experiment 1 was that it was not given immediately after participants’ response to the target which might have introduced unknown factors (sensory threshold variation at different times and under different circumstances) that could possibly have affected the test results. In Experiment 2, the visibility test was given immediately after participants responded to the target to minimize this possibility. A second purpose of Experiment 2 was to use the ISI as a within-subjects factor (in Experiment 1 it was a between-subjects factor) to rule out any possibility of a confound between ISI and group.

### Method

#### Participants

Twenty-two participants (14 women and 8 men) from China West Normal University volunteered for this experiment. All participants were right-handed, had normal or corrected-to-normal vision, and no history of, or current, neurological or psychiatric illnesses. They gave their written informed consent before the experiment and were paid for their participation. This study was approved by the IRB of China West Normal University.

#### Materials

The stimuli were the same as those in experiment 1.

#### Design and procedure

The experimental conditions and procedure were the same as in Experiment 1 (see Table 1 for details) with the following exceptions. The three ISI conditions (0 ms, 53 ms and 163 ms) were varied within-participants, the prime visibility was assessed within each trial, and the participants were required to take two prime visibility tests, a forced-choice discrimination task, and a PAS test (see Fig 1). In the PAS visibility tests, participants were first asked to decide whether the prime faces were of the same or different emotional expression by pressing “1” or “2” key. Then, participants reported on the quality of their subjective experience with the prime visibility on a 4-point scale using PAS: (1) ‘‘No experience”, (2) ‘‘Brief glimpse” (a feeling that something appeared but nothing more specific than that), (3) ‘‘Almost clear experience”, and (4) ‘‘Absolutely clear experience” (Atas et al., 2013; Szczepanowski et al., 2013). The choices of the visibility tests remained on the screen until the participant made a response.

#### Statistical analyses for assessing the prime-target congruence effects

The statistical analyses were the same as those of experiment 1 with two exceptions. First, only the correct responses to the targets associated with PAS ratings of “No experience” and “Brief glimpse” were included in analyses; second, the analyses were completely repeated-measures ANOVAs.

### Results

#### Prime visibility results

For the 0 ms ISI condition, the mean percentage of correct recognition was 50.41%, not significantly different from chance level, *t* (21) = .397, *p* = .696; nor was the mean d’ (mean = .029, SE = .091) significantly different from zero, *t* (21) = 0.322, *p* = 0.751. For the 53 ms ISI condition, the mean percentage of correct recognition was 49.05%, not significantly different from chance level, *t* (21) = -.792, *p* = .437; nor was the mean d’ (mean = .023, SE = .087) significantly different from zero, *t* (21) = .267, *p* = .792. For the 163 ms ISI condition, the mean percentage of correct recognition was 49.00%, again, not significantly different from 50%, *t* (21) = -.969, *p* = .344; nor was the mean d’ (mean = -.071, SE = .085) significantly different from zero, *t* (21) = -.836, *p* = .413.

In all the 66 blocks of PAS measures, there was no selection of “Almost clear experience” or “Absolutely clear experience”. The average percentage of ‘‘No experience” selection was 88.13% for the 0 ms ISI, 67.31% for the 53 ms ISI, and 77.23% for the 163 ms ISI condition, respectively. The average percentage of ‘‘Brief glimpse” selection was 11.87% for the 0 ms ISI, 32.69% for the 53 ms ISI, and 22.77% for the 163 ms ISI condition, respectively. The results again revealed that the prime faces were sufficiently masked when the same masking procedure was applied as in Experiment 1. The post-experiment queries revealed that some flashes of the prime before the target were judged as “brief glimpse”.

#### Overall congruency effect

The accuracy rates of the responses to the targets were submitted to a repeated measures ANOVA with valence relation in the prime (same versus different), valence relation in the target (same versus different) and ISI (0, 53, 163 ms) as within-participants factors. The ISI by prime valence relation by target valence relation 3-way interaction was not significant, *F* (2, 42) = .016, *p* = .984, η_p_^2^ = .001. The main effects of ISI, prime, and target valence relationship were all nonsignificant (all *Fs* < 0.595, all *ps* > .449). None of the interactions between prime and ISI, between target and ISI, and between prime and target was significant (all *Fs* < 3.669, all *ps* > .069).

**Fig 5:**
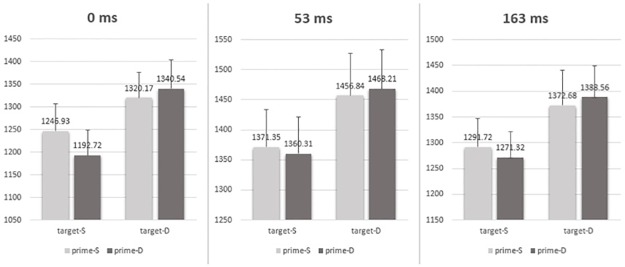
Mean RTs (ms) under each prime-target congruence condition (SS, SD, DS and DD) and each ISI (0ms, 53ms and 163ms). The results revealed a negative priming independent of three ISI conditions. Error bars represent the standard error of the mean.

A similar ANOVA with prime valence relation, target valence relation and ISI as within-participants factors was conducted on the data of the RTs for the correct responses to the target associated with the PAS ratings of “No experience” and “Brief glimpse” (see Table 2 and Fig 5). The ISI by prime valence relation by target valence relation 3-way interaction was not significant, *F* (2, 42) = 1.107, *p* = .340, η_p_^2^ = .050. The main effects of prime valence relation was not significant, *F* (1, 21) = 1.451, *p* = .242, η_p_^2^ = .065, whereas the main effects of target valence relation and ISI were significant, *F* (1, 21) = 31.576, *p* = .000, η_p_^2^ = .601 for the target, and *F* (2, 42) = 15.301, *p* = .000, η_p_^2^ = .421 for the ISI, respectively. The interaction between prime valence relation and target valence relation was also significant, *F* (1, 21) = 16.292, *p* = .001, η_p_^2^ = .437. But the interaction between prime valence relation and ISI, and the interaction between target valence relation and ISI were both nonsignificant (all *Fs* < 0.374, all *ps* > .690).

**Table 2:** The detail valence pairing information of each condition and the mean RTs±standard deviations (ms) as a function of ISI and prime-target congruence condition (overall congruency effect, visual and motor effects) in Experiment 2. For detail information, see Table 1.

Facial Emotional Valence Relation in Prime and Target. S = Same; D = Different.	SS		DD		DS	SD
	SS_s_	SS_c_	DD_s_	DD_c_		
Each Facial Emotion in Prime and Target. H = Happy; F = Fearful.	HH-HH(20)	HH-FF(20)	HF-HF(20)	HF-FH(20)	HF-HH(10)	HH-HF(10)
	FF-FF(20)	FF-HH(20)	FH-FH(20)	FH-HF(20)	HF-FF(10)	HH-FH(10)
					FH-HH(10)	FF-HF(10)
					FH-FF(10)	FF-FH(10)
0 ms ISI	1246.93±283.19		1340.54±293.99		1192.72±265.03	1320.17±258.60
	1245.20±278.85	1248.82±290.76	1348.11±306.12	1332.22±291.26		
53 ms ISI	1371.35±294.17		1468.21±307.52		1360.31±289.51	1456.84±331.08
	1340.22±308.25	1401.53±288.41	1469.08±327.18	1467.59±301.64		
163 ms ISI	1291.72±263.21		1388.56±287.23		1271.32±237.75	1372.68±320.23
	1297.04±267.58	1287.02±267.82	1381.94±273.57	1394.96±305.52		

The simple effects showed that (see Table 2 and Fig 5): (1) when the target was of different valence, the RT for the prime of different valence (mean = 1399.10 ms) was slower than for the prime of same valence (mean = 1383.23 ms), *F* (1, 21) = 6.23, *p* = .021; (2) when the target was of same valence, the RT for the prime of same valence (mean = 1303.33 ms) was slower than for the prime of different valence (mean = 1274.78 ms), *F* (1, 21) = 10.80, *p* = .004. In short, a negative priming was found and it was independent of ISI conditions.

#### Visual effect

**Fig 6:**
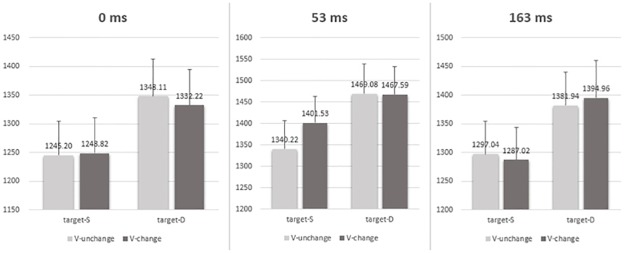
Mean RTs (ms) under each prime-target visual congruence condition (SS_s_, SS_c_, DD_s_ and DD_c_) and each ISI (0ms, 53ms and 163ms). The results did not reveal any visual effect. Error bars represent the standard error of the mean.

To investigate the visual effects, the RTs for the correct responses in which the PAS rating was “No experience” or “Brief glimpse” were submitted to a 3-way repeated-measures ANOVA with the valence relation between the two faces in the target (same versus different), visual feature change between prime and target (changed versus unchanged) (SS_s_ vs SS_c_; DD_s_ vs DD_c_) and ISI (0, 53, 163 ms) as within-participants factors (Table 2 and Fig 6). None of the interaction effects was significant (all *Fs* < 2.930, all *ps* > .102). The main effect of target valence relation and ISI were both significant, *F* (1, 21) = 26.635, *p* = .000, η_p_^2^ = .559 for target, and *F* (2, 42) = 12.844, *p* = .000, η_p_^2^ = .380 for ISI, respectively. However, the main effect of visual change from prime to target was not significant, *F* (1, 21) = 0.688, *p* = .416, η_p_^2^ = .032. These results suggested that the visual change from the prime to the target probably did not contribute to the above negative priming effect.

#### Motor response effect

**Fig 7:**
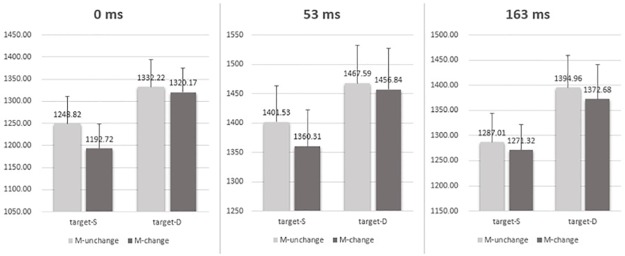
Mean RTs (ms) under each prime-target motor congruence condition (DS, SS_c_, SD and DD_c_) and each ISI (0ms, 53ms and 163ms). The results showed that the RTs to the changed response conditions (SD and DS) were faster than the RTs to the unchanged response conditions (DD_c_ and SS_c_) regardless of three ISI conditions. Error bars represent the standard error of the mean.

To investigate the motor response effect, the RTs for the correct responses in which the PAS rating was 1 or 2 were submitted to a 3-way repeated-measures ANOVA with the valence relation between the two faces in the target (same versus different), motor response changes between prime and target (SS_c_ vs DS; DD_c_ vs SD) and ISI (0, 53, 163 ms) as within-participants factors (see Table 2 and Fig 7). The main effect of target valence relation, motor response changes and ISI were all significant, *F* (1, 21) = 26.983, *p* = .008, η_p_^2^ = .562 for the target valence relation, *F* (1, 21) = 10.633, *p* = .004, η_p_^2^ = .336 for the motor response change, and *F* (2, 42) = 16.545, *p* = .000, η_p_^2^ = .441 for the ISI, respectively. None of the interaction effects was significant (all *Fs* < 0.620, all *ps* > .543). These results showed that the mean RTs to the response-changed conditions (SD and DS) were faster than the mean RTs to the response-unchanged conditions (DD_c_ and SS_c_) (see Fig 7**)**. In short, the motor effect indicated a negative priming effect, again, independent of ISI conditions.

## Discussion

By using a masked prime display paradigm, we investigated whether the emotional valence relation between two unconsciously perceived faces were processed by participants. The results showed that the unconsciously perceived valence relation in the prime elicited a negative priming effect independent of the length of ISI. Further analyses revealed that the negative priming effects were probably caused by the motor response effect. Overall, the results supported the idea of an integration (i.e., processing same-different valence *relation*) between two different unconsciously perceived stimuli. The findings are discussed in detail below.

The overall ANOVAs and simple effects tests in our two experiments indicated that the unconsciously perceived same/different valence relation in the prime influenced the accuracy and/or the RTs in response to the consciously perceived target to which participants made a same/different valence relation judgment. Our findings are consistent with the idea that unconscious processing could affect subsequent conscious responses as also reported in some other unconscious priming studies [35,36]. However, in previous studies, the unconscious information in the prime was derived mostly from a single stimulus. Recently, some studies started to investigate the integration between different unconscious stimuli in the prime. For instance, using a go/no-go task and simultaneously presenting two targets supraliminally and subliminally, Lin and Murray found that in the subliminal presentation condition (where participants made a go response) the RT to the paired same shapes was significantly faster than RT to the paired different shapes [10]. Also, it was found that the relation between multisensory (audiovisual) stimuli [9] and between two digits or two letters in the prime [11,12] could be processed unconsciously and influence the responses to the target. van Gaal et al. [28] also found that multiple unconsciously processed words could be semantically integrated as revealed in an EEG recording. The present study suggested that the subliminal same-different emotional valence relation in the prime was processed, as these valence relations were shown to affect the target processing. Similarly, in a recent study using ERP and fMRI techniques, Tu et al. found that two unconsciously perceived face pictures with different valences elicited a smaller N2 and increased activation in the left middle frontal gyrus compared with two unconsciously perceived face pictures with the same valence [27]. Tu et al. [27] considered this finding to reflect an unconscious detection of valence mismatch and suggested an integration between two unconsciously processed stimuli. The present study replicated the neural physiological effect with behavioral measures and further corroborated that hypothesis.

In the subliminal priming, a positive priming effect (faster response to a target preceded by a congruent prime than by an incongruent prime) normally appears when the ISI between the prime and the target is short but a negative priming effect (slower response to a target preceded by a congruent than an incongruent prime) emerges when the ISI is long [31,32]. The negative priming is generally thought to be caused by an inhibitory mechanism which suppresses the subliminal motor activation evoked by the prime [37]. A negative priming in our study refers to the finding that the RT to the target was slower when the valence relation in the prime was the same as that in the target compared with the condition in which the valence relation in the prime was different from that in target. Our simple effect analysis showed that the negative priming appeared to be unrelated to the ISI conditions. This finding contradicted the conventional view of a negative priming effect typically emerging under a longer ISI [32].

In the present study, the negative priming observed under the 163 ms ISI condition supported the above-mentioned inhibition view. However, a negative priming effect was also obtained under the 0 ms and 53 ms ISI conditions, which was seldom observed before. In Experiment 1, although RT priming effect was not observed when the two target faces were of the same valence, the response accuracy for the target was higher for the prime of different valence than for the prime of same valence which suggested a negative priming in response accuracy. In Experiment 2, the accuracy did not reveal any significant effect, but the RT showed a negative priming independent of ISI, regardless of whether the two target faces were of same or different valences. There were some variations in methodology between Experiment 1 and Experiment 2. The ISI was a between-subjects factor in Experiment 1 but a within-subjects factor in Experiment 2, and the visibility test was conducted at the end of the experiment in Experiment 1 but embedded within each trial in Experiment 2. These differences might be the sources of the slight differences in results between the two experiments. However, the similar results concerning the visual and motor effects between the two experiments overall supported the reliability of the results.

Because no pure visual effect was observed, we suggest that the negative priming in present study might not be attributable to the visual feature valence change from the prime to the target across the four conditions. The traditional view about the negative priming assumes that the transition from a positive priming to a negative priming as the target presentation is delayed might have been due to a contribution from a motor inhibition mechanism evoked during the delay interval [37]. In the present study, the valence relation in the prime was defined by the two masked faces. The negative priming effect observed under the 0 ms and 53 ms ISI might suggest that the negative priming depends not only on the duration of ISI, but also on the complexity of the prime and target stimuli (e.g. the relational integration) and the level of processing (e.g. emotional stimuli). In the present study, it is possible that the unconscious relation between the two prime emotional faces captured more attention or cognitive resources compared with a single stimulus prime. Although the relationship between attention and consciousness has been debated, some studies revealed that the attention can be deployed without consciousness [38–40]. There is also evidence that unconscious emotion stimuli can have an impact on attention [41–45]. Moreover, under the heavy attentional engagement with the prime in the present study, if the target valence relation was incongruent with the prime valence relation, it might be easier to escape from the attention capture evoked by the prime in this condition compared with the condition in which the target valence relation was congruent with the prime valence relation. Thus, an escape from attention capture could have led to a faster response to the target under the incongruent condition.

Another possible explanation for the negative priming at a short ISI is that the invisible emotional stimuli trigger rapid and automatic motor responses in short ISI, but traditional negative priming might be observable only at a long ISI when neutral stimuli are used [32,46]. There is evidence that a passive exposure to unseen facial or bodily expressions evokes faster facial reactions as recorded by electromyography and higher arousal as recorded by pupil dilatation than an exposure to consciously seen expressions [47]. This is consistent with the finding that physiological and behavioral responses evoked by unconscious emotional stimuli can be stronger and faster than neutral stimuli [48,49]. A transcranial magnetic stimulation (TMS) study also reported the influence of premotor area activities on the behavioral responses to unconscious perception of emotional faces [50], possibly because emotions prompt action tendencies. These two preliminary hypotheses need more investigation in the future.

As described in “Visual effect”, the visual effect, had it been obtained in the present study, should have been due to the valence alteration between the prime and the target, not the valence difference between the two masked faces in the prime. Therefore, a visual effect could not reveal the integration between the two masked faces. Further analyses indicated that motor effect might be the source of the negative priming in our study. The significant motor response change effect revealed that the RT to the motor response changed condition (DS and SD) were overall faster than the RT to the motor response unchanged conditions (SS_c_ and DD_c_) regardless of the duration of ISI. Because the visual effect was not observed, the motor effect in our study might have given rise to the negative priming effect.

Although no pure visual effect was observed in the present study, the visual effect was observed in a recent study in which a dissociation between the visual and the motor effect was obtained by manipulating the repetitions of a masked prime [29]. It seems that visual effects and the motor effects might coexist. Overall, the negative priming in the motor effect indicated that participants could process the valence relation in the prime and influence the responses to the target.

It should be noted that in the analyses of the visual effect in the present study, face pictures with the same valence were considered to be visually similar even though the two faces were of two different persons. It will be worthwhile to test a condition where identical face pictures are used and only the emotional expression is changed to investigate the visual effect. Also, the detailed mechanisms for causing the negative priming need more investigations in the future, for example, by using other types of stimuli. In addition, in the motor effect analysis, valence changed only on one face between the prime and the target in the DS and SD condition, each of which was supposed to contain both the visual and motor response change effects (e.g., FH-FF, HH-HF, respectively), whereas valence changed on both faces in the SS_c_ or DD_c_ condition, each of which was supposed to contain only the visual feature change effect (e.g., FF-HH, HF-FH, respectively). Therefore the motor effect analysis (SS_c_ vs DS and DD_c_ vs SD) possibly contained both visual and motor response change effects. However, because no pure visual effect was observed in the present study, we assume that the visual feature change should have played an insignificant role in the motor effect analysis.

Finally, there is an important issue in the study of unconsciousness that is still not completely settled. It is whether the stimuli of interest, i.e., the primes, are truly invisible at the conscious level. What is the best visibility test to use to insure the absence of consciousness is still under debate [51]. However, the strong masking and the results of the visibility tests conducted after the experiment as well as on each trial in the present two experiments should have given us reasonable assurance for the unconscious nature of the perception of the primes.

To conclude, in the present study we used complex facial emotional expressions as stimuli and provided behavioral evidence for the integration between subliminally perceived different facial emotional valences, i.e., high levels of unconscious information processing of facial emotional valence relation. This finding extended and confirmed the view that high-level information integration can take place without consciousness [13]. Recently, more and more studies have revealed that high-level information processing can take place at the unconscious level, including meaning integration between multiple words [28], between multisensory (audiovisual) modalities [9], between two digits or two letters [11,12] and between two shapes [10]. Also, our findings complemented those of Atas et al. [29] which indicated a visual but not motor response priming effect by suggesting that both visual and motor response congruence effects can possibly play a role in generating the priming effect. Importantly, a negative priming effect was found in the 0 ms ISI condition in our experiments. This negative priming finding contradicted the conventional view of the phenomenon and warrants further research for a better understanding of the mechanism underlying this phenomenon. Finally, the idea of the relationship between unconscious processes can have important theoretical implications for a new understanding of some cognitive and mental states and processes [7,8], such as a possible reinterpretation of the mechanism of creativity, a modification of global neural workspace theory, an integration of various consciousness theories, as well as new interpretations of resting states, sleeping, and even a redefinition of cognitive resources.

## Supporting Information

S1 Dataset SPSS data file showing raw RT data and related information for the participants. (ZIP)
